# How doctoral students’ role perceptions influence advisor-advisee relationships and academic progress: a case study

**DOI:** 10.3389/fpsyg.2025.1600872

**Published:** 2025-08-22

**Authors:** Hechun Wu, Jungyin Kim

**Affiliations:** Department of English Education, Jeonbuk National University, Jeonju, Republic of Korea

**Keywords:** role perceptions, expectation, behavior, advsior-advisee relationship, academic progress

## Abstract

**Introduction:**

This qualitative research investigated how doctoral (Ph.D.) students’ role perceptions influenced their expectations and behaviors, thereby influenced advisor-advisee relationships and academic progress. Doctoral advising is essential for doctoral students’ academic progress. One of the factors to influence doctoral students’ academic progress is the advisor-advisee relationships. Under the guidance of Biddle’s Role Theory, the researchers aimed to find out how doctoral students’ perceptions of the advisors’ roles and their own roles influenced their advisor-advisee relationships. Doctoral advisor-advisee relationship can influence doctoral students’ academic progress.

**Methods:**

By interviewing three Chinese Ph.D. students who studied in Korea, interview videos, emails, and messages between the participants and their advisors were collected as data. Data from the interviews were the main data resource. Other resources were used to support the data from interviews. These data were analyzed using qualitative methods, including transcription, coding, and member checking. The coding process was based on the transcription, and the member checking process ensured the validity the study.

**Results/findings:**

The findings indicated that variations in participants’ role perceptions can significantly influence the advisor-advisee relationships, which in turn influenced academic progress.

**Discussion:**

The patterns among the three participants showed that participants’ role perceptions influenced participants’ expectations and behaviors. Whether participants’ expectations were fulfilled, and whether participants’ behaviors were understood by the advisor influenced the advisor-advisee relationships. The advisor-advisee relationships influenced the participants’ learning experience and graduation, and further influenced the academic progress. Suggestions for advising Ph.D. students and limitations of this study were provided at the end of this study.

## 1 Introduction

Many universities face challenges in developing effective advising systems that enhance student satisfaction, foster positive learning experiences, and improve retention rates (Freeman, 2008). Academic advising has consistently been recognized as a critical factor influencing the success of doctoral students (Drake, 2011). Within the broader scope of academic advising, research has shown that the advisor-advisee relationship plays a fundamental role in doctoral students’ academic development (Parker-Jenkins, 2016). Thus, building and sustaining positive relationships between advisors and students are essential for supporting doctoral students’ academic progress. To achieve this, a deeper understanding of doctoral students’ expectations, their perceptions of advisors, and the motivations behind their behaviors is necessary (Anderson et al., 2014).

Previous studies (Kim and Feldman, 2011; Krase, 2007, etc.) predominantly treated students’ role perceptions, expectations, and behaviors as discrete, equally weighted factors influencing advisor-advisee relationships, while overlooking the underlying determinants of doctoral students’ expectations and behaviors. However, the role perceptions should serve as the foundational factor influencing the expectations and behaviors. According to Biddle’s (1979) role theory, various expectations and behaviors are determined by different role perceptions. Role perceptions can be viewed as a set of interrelated stereotypes that are constructed according to individuals’ experiences (Propp and Rhodes, 2006). Taylor and Crocker (1981) argued that personal stereotypes drive one’s impressions to fit his or her expectations. People’s behaviors can be influenced by role perceptions (Burke and Stets, 2009). Role perceptions can influence the expectations and the behaviors.

The primary purpose of this study was to investigate the factors influencing Chinese doctoral students’ advisor-advisee relationship in Korean universities, and the subsequent influence doctoral students’ academic progress. According to the Ministry of Education of South Korea, more than 40 % of the international doctoral students are from China in 2024. It is essential to find out the factors influencing Chinese doctoral students’ advisor-advisee relationship and academic progress in Korea context. After primary data collection and based on empirical data analysis, it was found that students’ expectations and behaviors emerged as the predominant influencing factors. Further analysis revealed that doctoral students’ role perceptions constituted the fundamental determinant shaping their expectations and behaviors. Consequently, this study focus shifted to examining how doctoral students’ role perceptions influenced the advisor-advisee relationship and the academic progress.

The researchers believe that variations in doctoral students’ role perceptions lead to different expectations and behaviors, which can influence the advisor-advisee relationship and further influence students’ academic progress. Therefore, it is important to explore how Ph.D. students’ different role perceptions influence the advisor-advisee relationship, and how the advisor-advisee relationships influence students’ academic progress. Accordingly, two research questions are addressed:

1. In what ways do doctoral students’ role perceptions shape their engagement in advisor-advisee relationships?
2. How do these engagements facilitate or hinder academic progress?

Through addressing the research questions, this study systematically examined how variations in doctoral students’ role perceptions, which can influence doctoral students’ advisor-advisee relationship and doctoral students’ academic progress. The study further investigated the determinants of positive/negative advisor-advisee relationships, with the ultimate objective of enhancing doctoral students’ academic progress through optimized advisor-advisee relationships.

## 2 Literature review

### 2.1 Role theory and role perceptions

Role perceptions are cognitive beliefs that can change in response to changes in the external environment or within the person (Grant and Hofmann, 2011). Consequently, role perceptions demonstrate inter-individual variability across distinct environmental contexts and intra-individual divergence within identical settings. Role perceptions can influence expectations and behaviors. A central argument of role theory is that what individuals expect and behave is determined by how they identify their roles (Biddle, 1979). Concepts such as role conflict, role ambiguity, and role negotiation were introduced in Biddle’s (1979) Role Theory, which can explain how the role perceptions constructed, maintained and negotiated. On the one hand, individuals’ role perceptions determine their expectations to a certain degree (Taylor and Crocker, 1981). On the other hand, there is a match between a role and behavior (Sluss et al., 2011). People behave differently based on their different role perceptions (Burke and Stets, 2009). In summary, people’s role perceptions can influence their expectations for others and behaviors.

### 2.2 Advisor-advisee relationships in doctoral education

The advisor-advisee relationships between Ph.D. students and their advisors can be influenced by students’ expectations of the advisor and students’ behaviors. Eby and Lockwood (2005) identified that advisors’ failure to meet advisees’ expectations created the most commonly reported problems. Young and Perrewe (2000) found that fulfilling expectations mediated the relationship between advisors and advisees, which can bring advisors’ support and trust. Understanding students’ expectations of academic advising was the most imperative step in improving the quality of academic advising (Kim and Feldman, 2011). Previous studies found that fulfilling doctoral students’ expectations was essential to maintain positive advisor-advisee relationships. In Krase’s (2007) study, one of the reasons that affected the advisor-advisee relationship was that the advisor could not understand the advisee’s behaviors. Lack of communication was why the advisor could not understand the advisee’s behaviors, which led to a negative advisor-advisee relationships (Krase, 2007). It showed that understanding students’ behaviors was essential to maintain positive advisor-advisee relationships as well. Both fulfilling expectations and understanding students’ behaviors were considered as two key factors to maintain a positive advisor-advisee relationships. However, previous studies only considered students’ fulfilling expectations and understanding students’ behaviors as separated factors and did not further explain the factor influencing the expectations and behaviors.

Advising was at the heart of the institutional and interpersonal structures that make up graduate education (Chapman and Sork, 2001). Therefore, the relationships that doctoral students developed with their advisors was crucial to their success in completing their graduate degree (Barnes, 2009). De Valero (2001) reported that doctoral students who developed a positive relationship with their advisors had shorter times to degree completion. Doctoral advisors’ social support can significantly influence students’ graduation rate (Jairam and Kahl, 2012). Graduation, as one of the main targets for doctoral students, can be influenced by advisor-advisee relationships. Previous studies showed that doctoral students’ learning experiences were related to retention rate. Grasky (2018) uncovered positive connections within advisor-advisee relationships related to social and academic matters. Both Joslin (2018) and Tinto (2012) warned that poor learning experiences can affect a student’s satisfaction with an institution. Thus, maintaining positive advisor-advisee relationships is important for Ph.D. students’ academic progress, persistence of doctoral study, Ph.D. students’ learning experiences, and graduation.

### 2.3 Cultural context: Korean higher education and international students

According to the official website of the Ministry of Education of South Korea, doctoral education in South Korea saw its R&D (Research and Development) investment accounting for 4.92% of the GDP in 2025. Moreover, the annual growth rate of university research funds reached 7.3%. The proportion of international students has exceeded 34%, and the coverage rate of English-taught programs has increased to 77% by 2025. The standard academic duration of doctoral study in Korea is 3 years. The doctoral training process is divided into three stages: course study, comprehensive test, and oral defense. Academic output requirements of arts speciality, such as English Department, are two journal papers of KCI or one SCI/SSCI. Ignorance of cultural differences can cause misunderstandings and misinterpretations, that can result in unintended insults and disputes (Steers and Osland, 2020). Power distance is relatively high in Korea, and it means that inequality is found in Korean society (Kim, 2025). International students may experience psychological stress due to issues related to Korean culture of “generational etiquette (선후배)” (Kim, 2025). Senior students have more decision-making power and speaking rights than junior students. High-context communication can be found in Asian countries, such as Korea (Chen et al., 2025). The way of speaking can be indirect and it is crucial to look at body language, facial expression, and other social cues that might indicate the true meaning (Steers and Osland, 2020). International students may misunderstand the true meaning when communicate with Korean because of high-context communication. Galinova (2014) suggested to establish a cultural sensitivity communication framework to reduce the risk of misunderstandings arising from high-context cultures.

## 3 Methods

The primary purpose of this study aimed to identify the factors influencing doctoral students’ advisor-advise relationship. Preliminary investigations revealed that students’ expectations and behaviors constituted the principal factors affecting advisor-advisee relationships. In order to further investigate what influenced students’ expectations and behaviors, the researchers found that Biddle’s Role Theory was adopted as the theoretical framework. Individuals may identify their roles differently, and different role perceptions in individuals’ minds can influence their expectations and behaviors. Thus, doctoral students may have different role perceptions of the advisors’ roles and their own roles, which can influence their expectations for the advisors and the behaviors to further influence the advisor-advisee relationship and the academic progress. Accordingly, the study’s methodological approach (including research design, data collection protocols, and analytical strategies) was specifically tailored to examine role perception constructs.

### 3.1 Context

This study was conducted in the English Language and Literature Department at a Korean university. English was the language that professors usually used to teach and communicated with international Ph.D. students. There was no Korean doctoral student in the English Department in this university from 2020 to 2025. The three research participants have chosen the same male professor as their advisor. This professor’s courses for Ph.D. students were “one-on-one” courses, meaning the professor would teach one student at a time. This professor’s job included both teaching and advising. The teaching part was for all the doctoral students who chose his courses. The professor would not teach all the students at once, and the students were supposed to meet the professor one by one. One course would last 30 min for each student per week and occur in the professor’s office. The other part of this professor’s job was advising the students who chose him as advisor. After students chose the professor as advisor, the professor would ask students to collect data and write the journal paper and final dissertation according to the professor’s advising. The “one-on-one” courses would be changed to advising, which was still “one-on-one” advising and lasted for 30 min.

The participants described their advisor as a scholar under 50 years of age who had earned his doctoral degree in the United Kingdom. When this advisor talked in Korean with locals, he behaved like a Korean. When this advisor talked in English, this advisor behaved similar to English native speakers. It might be the advisor’s international studying experience influenced his behaviors. The participants were professors in English Department for years who can fit the advisor’s behaviors because they communicated with the advisor in English. Besides, it was not the first time that the advisor advised Chinese doctoral students. The advisor can understand that the participants were Chinese who might not quite familiar with Korean culture, and the advisor tried to avoid the culture conflict. Participants reported minimal perceived cultural conflicts during the communication with the advisor.

### 3.2 Participants

The three participants- Katherine, Alice, and Quinn (all fake names) - were one of the researchers’ close friends. In qualitative researches, establishing researcher-participant rapport is essential for data authenticity. Since one of the researchers have already built the connections, the participants can honestly express their thoughts and stories. The reason why the researchers selected three participants was because of the saturation of the data collection and analysis. Selecting three participants would be suitable to describe each participant’s experiences in the case study. The participants were mainly selected according to their similar backgrounds. First, all three participants are Chinese and have similar cultural backgrounds, which ensured that students’ cultural backgrounds would not influence the advisor-advisee relationships and academic progress. Second, all the participants chose the same advisor in the English Department at one university. The influence from different advisors’ advising, such as different advising styles, toward the participants was controlled. Thus, the participants’ advisor-advisee relationships and academic progress would not be influenced by their different advisors. Third, all the participants are professors and advisors themselves in China. The participants have the experience of advising students when they were professors. As advisors, all of the participants advised undergraduate students independently, and they need to guide more or less ten students to complete the dissertation every year. The participants’ advising experiences ensure that they clearly perceive their role and the advisor’s role in the advisor-advisee relationships. The participants’ profiles are listed in Table 1.

**Table 1:** Participants’ profile.

Name	Age	Gender	Nationality	Major	Graduated or not until 2025	Years of advising in china
Katherine	38	female	China	English Linguistics	yes	7
Alice	49	female	China	TESOL	yes	14
Quinn	52	female	China	English Linguistics	no	16

There were disadvantages to select the participants with similar backgrounds. The participants’ gender and the advisor’s gender relatively limited the founding of this study. The researchers tried to involve male participants, only one of the male students who followed a male advisor promised that he would join. However, after two interviews, this male student dropped. Another limitation of this study is that the advisor was not included as a participant. The researchers also tried to invited the participants’ advisor as a participant. However, the advisor refused. The researcher can only estimate the advisor-advisee relationship according to the advisor’s behaviors toward the students. The advisor’s behaviors were described by the participants. The participants’ description were supported by additional resources such as emails and messages between the advisor and the participants. Thus, the interpretation of the advisor’s behaviors can be considered as valid.

### 3.3 Data collection and analysis

Data collection was conducted from August 2023 to December 2024. The data were mainly collected via semi-structured interviews. The participants were individually interviewed, and each interview lasted from 30 to 90 min. The interviews were conducted in Mandarin to minimize potential meaning distortion. The common semi-structured interview questions related to the key concepts in this study were listed in the appendix (Appendix: Semi-structured interview questions). All the interviews were conducted online and video-recorded by software. Thus, the researchers could watch and listen to the interviews multiple times with each participant’s facial expression. Additional resources were emails and text messages between the participants and the advisor. Due to the reason that it was difficult to observe the live communication between the participants and the advisor, emails and text messages made up for this defect to a certain degree. In order to avoid researchers’ bias, the collected data and data analysis were checked by the colleagues who were not involved in this study as peer review. Most of the emails and text messages were only shown to the researchers to support the participants’ description of the advisor’s behaviors and the advisor-advisee relationship. The researchers tried to avoid showing the emails and text messages due to the ethical considerations. Besides the interviews for the participants, there was one interview for a peer professor in the same department and university. The purpose of interviewing this professor was to confirm the participants’ advisor can not interpret Alice’s intention of asking for feedback. Different data types allowed for cross-checking, which improved the validity of the research.

Data analysis followed a three-phase grounded theory approach: transcription, coding, and member checking. During transcription, the researchers systematically documented participants’ narrated experiences regarding advisor-advisee relationship and academic progress through repeatedly video analysis. This phase established the foundational narrative framework for subsequent analysis. The coding process was based on grounded theory, which included open coding, axial coding and selective coding. Open coding was based on the transcription to collect meaningful units. Axial coding was based on the open coding to connect the meaningful units, such as causal relationship between the two meaningful units. Selective coding was based on the axial coding to merging the connections of the meaningful units. One of the researchers was responsible for the coding process. The other researcher who had participated in multiple qualitative studies was responsible for checking the results of the coding. The two researchers’ cooperation guaranteed the reliability of the coding process. A sample of coding process is showed in Table 2. This step helped the researchers confirm that participants’ role perceptions were the main factors that influenced the advisor-advisee relationship, and academic progress. At last, the researchers sent the findings to the participants, respectively, for member checking. This step validated the accuracy of the researchers’ interpretation.

**Table 2:** Sample of coding process (Katherine).

Raw data	Transcription	Open coding	Axial coding	Selective coding
*When I was writing my dissertation, I suffered on analyzing my data for weeks without finding a good result. However, I did not try to find my advisor to ask for help, because I knew my data more than my advisor* (Translated Interview for Katherine, September 6th, 2023).	Katherine solved her data analysis problem by herself and did not expect or rely on the advisor’s help, because she thought the she knew more.	independent, low expectation, taking in charge (meaningful units)	Katherine’s role of first person in charge influenced her low expectation which was easy to be fulfilled (causal relationship)	Katherine’s role of first person in charge led to easily fulfilled expectation which influenced the advisor-advisee relationship (connections)
*… You cannot expect the advisor to explain everything clear for you. Advisors only need to provide some hints… If you do not understand but interested, you need to search it yourself* (Translated Interview for Katherine, April 19th, 2023).	Katherine did not expect much from the advisor. The advisor only need to provide hints. Katherine can learn more by herself.	low expectation, independent (meaningful units)	Fulfilled expectation influenced the advisor-advisee relationship to be positive.	
*Our relationship was not very closed, but good and comfortable for me* (Translated interview for Katherine, September 6th, 2023).	Katherine was comfortable to get along with the advisor.	positive relationship (meaningful units)	positive relationship	

### 3.4 Researchers’ role

One of the researchers who collected data was a close friend of the three participants. The role of a friend provided the participant’s comfortable environment and trust during the interview. The participants were willing to share their personal stories and anecdotes about the advisor-advisee relationship and academic progress. The participants came to Korea earlier than the researcher. The participants also saw the researcher as a junior fellow student, which allowed the participants to pass their learning experience as seniors. Besides, one of the researchers and the participants were all English major doctoral students, which made the researcher and the participants had more common topics as colleagues. More common topics created an understanding environment for the researcher and the participants. In general, the researcher’s roles as a friend, a junior fellow student, and a colleague to the participants ensured the depth of the data.

## 4 Findings

This section explored how participants’ different role perceptions influenced the advisor-advisee relationships and their academic progress. The representative data and analysis showed each participant’s perceptions and experience with the advisor, which can influence the advisor-advisee relationships and their academic progress.

### 4.1 Katherine

#### 4.1.1 How was the advisor-advisee relationship influenced by Katherine’s role perception

Katherine’s perception of her advisor’s role was an “assistant,” and Katherine herself was the “first person in charge.” Katherine stated that her advisor’s function was to assist during her doctoral study (Interview for Katherine, October 27th, 2024). According to Biddle (1979), individuals have understandings of their roles and others’ roles. Katherine’s role perceptions were the roles of the advisor and herself in her mind. In reality, the advisor can not be on the position of an assistant, and Katherine did not have the power to be the first person in charge. Katherine used the metaphor to describe how she understood the relationship between the advisor and herself. Katherine’s understanding would guide her expectations and behaviors. According to Katherine, her perceptions of her role and her advisor’s role were mainly influenced by the trust and respect of her parents. Since Katherine was a child, her parents supported her in making her own decisions and dealing with her own problems independently (Interview for Katherine, October 27th, 2024). When it came to Katherine’s doctoral study, Katherine also considered it as her own matter.

Because of Katherine’s role perceptions, Katherine had relatively low expectations for the advisor. When the researcher asked Katherine’s expectations for the advisor, Katherine answered:

*My expectation for the advisor was easy to communicate. An advisor who did official business according to official principle and did not intentionally giving me a hard time was good enough for me. When I was writing my dissertation, I suffered on analyzing my data for weeks without finding a good result. However, I did not try to find my advisor to ask for help, because I knew my data more than my advisor* (Translated Interview for Katherine, September 6th, 2023).

Katherine’s definition of “easy to communicate” was that the advisor should request Katherine according to the university’s graduation requirement without excessive requests and extra pressure. In Katherine’s perspective, she should not rely on the advisor. The advisor was considered to “know less” than Katherine herself in her research. When Katherine met difficulty, she would rather find the solution herself than look for a solution from her advisor. Katherine’s low expectation for the advisor was influenced by Katherine’s role perception.

Katherine’s low expectations for the advisor influenced the advisor-advisee relationship. Katherine stated an example of when she felt her advisor was helpful:

*Once, my script (a program to analyze data) cannot run. I met my advisor and wanted him to solve the problem for me. Our meeting time should be half hour, but he (advisor) checked for about fifty minutes. He checked it for me line by line, back and forth. Even, at last, he did not find the problem, I still felt appreciate for his time and help* (Translated interview for Katherine, May 9th, 2024).

Katherine’s expectation for the advisor was to solve her script problem. The advisor tried to help but failed. However, because the advisor was willing to spend his own time, and tried his best to help, Katherine still felt that she was helped. Because of Katherine’s low expectations for her advisor, the help from her advisor was considered appreciated instead of disappointed. This thought helped Katherine establish and keep a relatively good relationship with her advisor. As Katherine described, “Our relationship was not very closed, but good and comfortable for me” (Interview for Katherine, September 6th, 2023). Thus, Katherine’s role perception led to Katherine’s low expectations of the advisor, which helped Katherine maintain a good advisor-advisee relationship. Katherine’s advisor-advisee relationship was mainly influenced by Katherine’s own role as the “first person in charge.” In Propp and Rhodes’s (2006) study, the students’ perceptions of the advisors’ roles were found to influence the advisor-advisee relationship. However, the students’ perceptions of their own roles should be considered as well. Thus, both of students’ role perceptions of the advisors and students need to be considered.

#### 4.1.2 How was Katherine’s academic progress influenced by the advisor-advisee relationship

Kathrine’s academic progress was smooth because of the good advisor-advisee relationship. Her learning experience during her academic progress was described as smooth and with little pressure (Interview for Katherine, April 27th, 2024). According to Kathrine, the learning experience during her academic progress was essential. Kathrine stated her learning experience as follows:

*The good (advisor-advisee) relationship made us understand each other’s behavior. I was a person who focus on experience. If I did not have good relationship with my advisor, I had to meet an advisor who made me feel nervous and anxious every time. It was not good for my health* (Translated interview for Katherine, April 27th, 2024).

This statement indicated that Katherine felt that her advisor and she understood each other, which provided her positive learning experience during her doctoral study. High levels of anxiety often impairs concentration and the ability to remain on task (Sadock and Sadock, 2000). Katherine considered her academic progress was smooth because she had no anxiety. The understanding between advisor and advisee led to little pressure and worries, which provided Katherine positive learning experience with emotional well-being. Katherine considered that a positive learning experience was related to her study and health, which was important for her academic progress. The reason why she had a positive learning experience was that she had a good relationship with her advisor. In a supportive environment (Buirski, 2022), doctoral supervision can lead to comprehensive research outcomes and produce well-prepared scholars to contribute significantly to their respective fields. Thus, a good advisor-advisee relationship provided Katherine positive learning experience, which influenced Katherine’s academic progress.

In summary, for Katherine, it showed the answer to research question one that Katherine’s role perception influenced her expectations which influenced the advisor-advisee relationship. The advisor-advisee relationship in turn led to the results of Katherine’s positive learning experience and smooth academic progress for research question two.

### 4.2 Alice

#### 4.2.1 How was the advisor-advisee relationship influenced by Alice’s role perception

Alice’s perception of her advisor’s role was a “general,” and Alice herself was a “soldier.” A general was in a position to provide guidance, and a soldier was in a position to follow guidance (Interview for Alice, October 1st, 2024). Alice’s role perceptions were the roles of the advisor and herself in her mind. In reality, the advisor was not a general, and Alice was not a soldier. Alice used the metaphor to describe her understanding of the relationship between the advisor and herself. Alice’s understanding would guide her expectations and behaviors. Alice materialized the guidance from an advisor by providing feedback, which was also the advisor’s duty (Interview for Alice, April 14th, 2024). Alice’s role perception was mainly influenced by her working experience as a professor in China. When Alice worked as an advisor herself, she considered that she should be the one who led her students and consistently provided feedback without students’ requests (Interview for Alice, July 22nd, 2024). Thus, Alice expected that her doctoral advisor could provide feedback without Alice’s requests.

**Figure 1:**
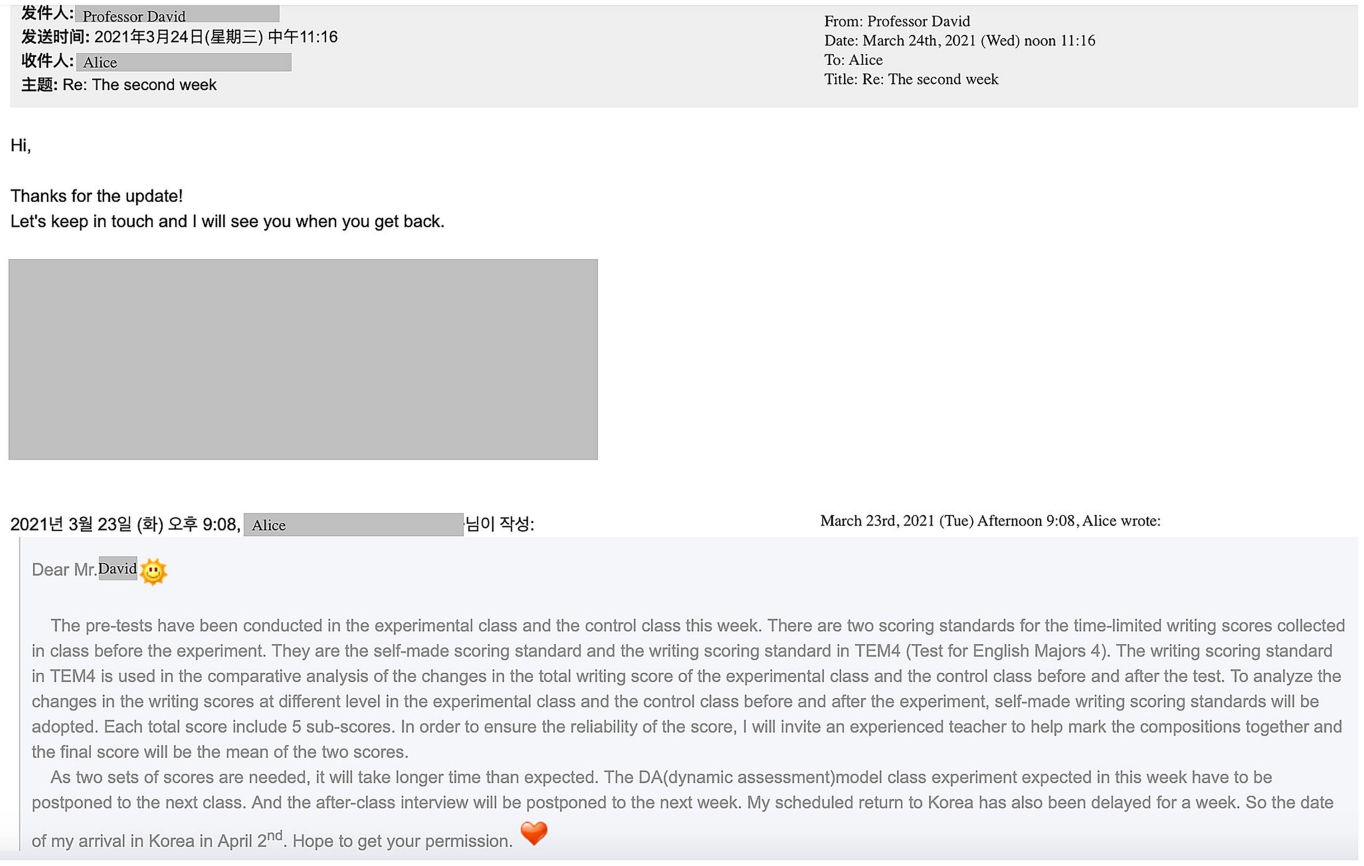
Email between Alice and her advisor.

Alice ended the advisor-advisee relationship with her previous advisor. Alice explained the reason as: the advisor never replied to her emails (Interview for Alice, July 6th, 2024). An example email between Alice and her previous advisor is shown in Figure 1.

In this email, the previous advisor replied. It was contrary to what Alice stated. So, the researcher asked Alice why she considered her email was not replied. Alice stated that her intention in sending the email was to ask for feedback (Interview for Alice, July 6th, 2024). However, the advisor did not provide any feedback. Alice’s expectation of getting the advisor’s feedback was not fulfilled. Alice saw the previous advisor as having no value to her academic improvement. So, Alice decided to end the advisor-advisee relationship and change to another advisor.

This email did not show that Alice directly asked for feedback. In order to confirm that the researcher’s assumption of “no sign of asking for feedback” was correct, the researcher showed this email to a professor in the same department and university as Alice studied. This professor confirmed that he can not interpret any sign of asking for feedback (Interview, Oct 7th, 2024). When two professors from the same department can not interpret Alice’s intention, it reflected Alice’s intention was not obviously expressed. The researcher pointed out to Alice that there was no sign of asking for feedback in this email. Alice answered,

*Why did I send him emails about my work? Because he was my advisor and leader, and he had duty to guide me… Maybe I did not say it out, but did I need to say everything so clear?… An advisor should check the content and point out what should be fixed* (Translated interview for Alice, July 6th, 2024).

In Alice’s perspective, it was the advisor’s responsibility to provide feedback to students, and students did not have to ask for it. Gruzdev et al. (2020) and Wang and Parr (2021) argued that doctoral research work is often seen as a journey during which students receive guidance from their advisors. Thus, Alice considered that the previous advisor was not qualified. Burgoon (1993) found that when behaviors displayed by advisors violate what students expect, these violations can damage future interactions. In this study, it was Alice that did not request the feedback from the advisor. The advisor might not or can not notice Alice’s expectation. The source of the problem stared from Alice’s indirect expression of her expectations. The expectation ambiguity of Alice mainly caused the advisor’s replying the email without feedback. However, in Alice’s perception, the previous advisor can not fulfill Alice’s expectation. As a result, Alice ended the advisor-advisee relationship and changed to another advisor. The advisor-advisee relationship was influenced by Alice’s role perceptions.

#### 4.2.2 How was Alice’s academic progress influenced by the advisor-advisee relationship

Alice’s graduation was delayed for one semester because she got an “F” on her previous advisor’s course. After Alice changed advisor, Alice went back to China to collect data before winter vacation. When Alice returned to Korea, she saw an “F” on her previous advisor’s course. Alice found out she got an “F” because she returned to China before winter vacation. Alice considered this event to happen because of changing her advisor. Alice stated her explanation of her consideration as follows:

*I was not the only one who went back to China before winter vacation in that semester. Katherine also went back which was same as me. But Katherine did not got an “F” on this professor’s course.* (Translated interview for Alice, March 17th, 2024).

*Before I changed advisor, I got straight “A^+^” on his courses. But after I changed advisor, I got an “F”…. It was professor’s attitude problem toward me because I changed advisor* (Translated interview for Alice, June 6th, 2024).

Depending on Alice’s explanation, Alice and her peer classmate returned to China before winter vacation in the same semester. However, only Alice got an “F.” Alice got a straight “A+” before, but she got an “F” after she changed advisors. The previous advisor treated Alice differently from other students and graded Alice differently from before. It was reasonable to consider that the action that Alice ended the advisor-advisee relationship with her previous advisor has influenced Alice’s marks. When Alice failed one course, she had to take the comprehensive test in the following semester. This was the reason why Alice’s graduation was delayed for one semester. Therefore, Alice’s advisor-advisee relationship led to her delayed graduation and Alice’s negative learning experience, which influenced Alice’s emotional well-being and academic progress. Universities struggle to develop and maintain effective advising services to increase retention rate (Freeman, 2008). Even though Alice did not drop the doctoral program, it was possible for Alice to drop the program because of her negative learning experience and delayed graduation. Thus, doctoral students’ learning experience and delayed graduation need to be considered as factors to influence students’ academic progress.

Thus, for Alice, it showed the answer to research question one that Alice’s role perception influenced her expectations and behaviors which influenced the advisor-advisee relationship. The advisor-advisee relationship further led to the results of Alice’s negative learning experience and delayed graduation for research question two.

### 4.3 Quinn

#### 4.3.1 How was the advisor-advisee relationship influenced by Quinn’s role perception

Quinn’s perception of her advisor’s role was “experienced researcher,” and Quinn herself was “novice researcher” (Interview for Quinn, November 9th, 2024). Quinn’s role perceptions were the roles of the advisor and herself in her mind. In reality, the advisor should be on a higher position than a co-researcher. However, in Quinn’s mind, her understanding of the relationship between the advisor and herself was that they were co-researchers. Quinn’s understanding would guide her expectations and behaviors. Quinn’s perception was mainly influenced by her research experience as a co-researcher. As a professor in China, Quinn had experience doing researches with other researchers, which gave Quinn the impression that doing research with her advisor should be the same as doing researches with other researchers (Interview for Quinn, December 19th, 2024).

Quinn had expectations to be treated as a co-researcher. Quinn was invited to a research project by her advisor in April 2024. In order to join in the research, Quinn changed her plan of finishing her final dissertation and focused on the research. In the research, Quinn was mainly asked to find participants and collect data. However, the detailed research design were not told to Quinn. Quinn tried to ask for this information, but her advisor evaded Quinn’s questions and asked Quinn to follow the lead. Quinn complained in the interview:

*My advisor did not want to tell me details, and only wanted me to collect data… When I asked questions about the experiment design, he told me to skip the experiment design, and only provided me several steps and notices to do the experiment* (Translated interview for Quinn, July 20th, 2024).

In Quinn’s perspective as a co-researcher, she had the right to know the details. Quinn felt disappointed about the advisor. Quinn’s expectation of being a co-researcher was not fulfilled. There was role misalignment in Quinn’s case. Quinn saw herself as a co-researcher might not fit the role as a student in reality. Bahtilla and Oben (2022) emphasized the importance of effective communication between students and advisors to share understanding, address uncertainties, and establish clear expectations. However, the advisor avoided sharing information with Quinn. It was why Quinn complained about her advisor, which influenced the advisor-advisee relationship.

Quinn’s role perceptions influenced her behaviors as well. Quinn modified the experiment design without communicating with her advisor. The advisor was angry that Quinn did not strictly follow the advisor’s instruction, and called off the cooperation with Quinn in June, 2024 (Interview for Quinn, November 9th, 2024). Quinn stated the reason of her behavior of modifying experiment design as follows:

*He asked me to do the experiment, and he would analyze the data. I considered that I can modify the experiment design because I was the researcher who actually did the experiment… I thought that I could do the experiment first, and talk to my advisor latter. If this changing of collecting data online worked, it would benefit a lot for the following of this research…* (Translated interview for Quinn, November 9th, 2024).

Quinn’s behavior in modifying the experiment design was influenced by her role perceptions. As one of the researchers, Quinn considered that she could modify the experiment design without getting the advisor’s permission first. Quinn did not notice that she might not have the power to modify the research design. When Quinn considered the modifying would be benefit for the research, she just modified. The advisor was angry at Quinn about the changing experiment design and called off the cooperation, which affected their advisor-advisee relationship. Quinn’s modifying the research design without communicating with the advisor mainly caused the advisor’s anger and calling off the cooperation. Quinn’s behavior can be considered as hierarchical deferral, and the hierarchical deferral was caused by Quinn’s role perceptions. Thus, Quinn’s role perceptions influenced her behavior, which influenced the advisor-advisee relationship. Previous studies, such as Anderson’s et al. (2014) study and Fullick et al.’s (2013) study, mainly focused on the fulfillment of students’ expectations to increase academic advising. In this study, Quinn’s story showed that understanding students’ behaviors should be considered as another aspect to increase academic advising.

#### 4.3.2 How was Quinn’s academic progress influenced by the advisor-advisee relationship

After Quinn’s advisor called off the cooperation with Quinn, the advisor-advisee relationship became intense. Quinn tried to send emails and messages to apologize for her behavior. However, the advisor did not reply (Interview for Quinn, November 9th, 2024). After Quinn finished her final dissertation draft, Quinn sent her advisor an email, but the email was not replied to either (Interview for Quinn, November 9th, 2024). Quinn stated her situation as follows:

*Since the time he was angry at me, he has not replied my emails and messages of apologizing and holiday blessing. In last month, I sent him the draft of final dissertation, there was no reply either. I can only try to revise my final dissertation myself for now* (Translated Interview for Quinn, November 9th, 2024).

According to Quinn, when she and the advisor worked on the research, they met weekly, and the advisor replied to her emails (Interview for Quinn, July 20th, 2024). Because of the intense advisor-advisee relationship, the communication between Quinn and her advisor became less. Quinn did not get the suggestions for dissertation revision from the advisor, which influenced Quinn’s learning experience and delayed Quinn’s graduation. Quinn’ learning experience was related to her emotional well-being. Quinn felt disheartened when the advisor avoided contacting her (Interview for Quinn, November 9th, 2024). Quinn stayed in this doctoral program for ten semesters until 2025, and she did not graduated. Quinn may drop the program if her situation of not being able to graduate persisted. Thus, Quinn’s negative learning experience and delayed graduation influenced Quinn’s academic progress.

To sum up, for Quinn, it showed the answer to research question one that Quinn’s role perception influenced her expectations and behavior which influenced the advisor-advisee relationship. The advisor-advisee relationship further led to the results of Quinn’s negative learning experience and delayed graduation for research question two.

The following table was provided to clarify the patterns across the three cases. It reflected that the participants’ role perceptions influenced their expectations and behaviors. When the participants’ expectations were fulfilled/not, and the behaviors were understood/not, the advisor-advisee relationships tended to be positive/negative. The advisor-advisee relationships influenced the participants’ learning experiences and graduation (Table 3).

**Table 3:** Patterns across the cases.

Participants	Katherine	Alice	Quinn
Advisor’s role	assistant	general	experienced researcher
Their own roles	first person in charge	soldier	novice researcher
Expectations for the advisor	“easy to communicate”	feedback	sharing information
Expectation fulfilled/not	fulfilled	not fulfilled	not fulfilled
Students’ Behaviors	appreciated with the help	changed advisor	modified experiment design
Advisor’s response	tried the best to help	grading “F”	avoided contacting
Behaviors are understood/not	yes	no	no
Advisor-advisee relationships	positive	negative	negative
Learning experiences	positive	negative	negative
Graduation	not delayed	delayed	delayed

## 5 Discussion

**Figure 2:**
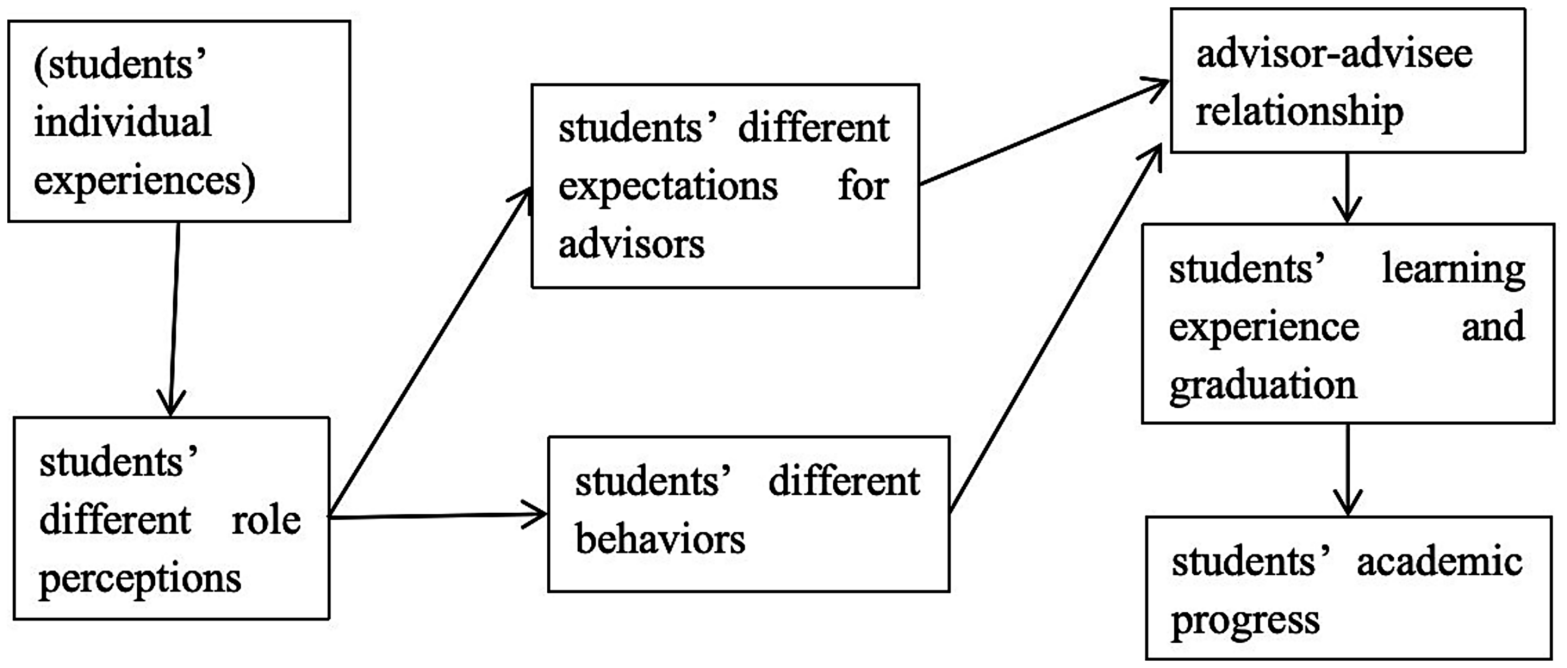
Framework.

This section addressed the two research questions by examining the mediating effects of role perceptions on the advisor-advisee relationships and the academic progress. It was suggested that participants’ different role perceptions influenced participants’ expectations for their advisors and participants’ behaviors, which can influence the advisor-advisee relationship. The advisor-advisee relationship influenced students’ learning experience and graduation, which can influence students’ academic progress. Figure 2 shows the framework that how students’ role perceptions influenced the advisor-advisee relationship and the academic progress.

### 5.1 Research question 1: in what ways do doctoral students’ role perceptions shape their engagement in advisor-advisee relationships

In this research, all three participants had different perceptions of their own roles and their advisor’s role during their doctoral study. One of the reasons that led to different perceptions was that the perceptions were shaped by past experiences (Robbins and Judge, 2017). The participants’ role perceptions might be influenced by Chinese culture as well. The influence of the culture was through how the participants were raised up, were educated, and learned from others’ behaviors in the culture. Thus, the participants’ Chinese culture experience can be considered as the participants’ past experience. In this study, the main reason for the participants’ different role perceptions was the past experience. While the participants’ role perceptions were constructed based on their past experiences, there should be role negotiation during their doctoral study in the Korean university. However, after the role negotiation, the participants mainly maintained the role perceptions influenced by their past experiences in China. Maybe, they considered their past experiences can solve the problems during doctoral study.

When the participants had different perceptions of their roles and the advisor’s role, they had different expectations for the advisor and proceed with different behaviors.

#### 5.1.1 Expectations

Students’ different role perceptions influenced students’ expectations. Propp and Rhodes (2006) found that students’ mental construct of the advisor’s role guides students’ expectations of advisors. In the current research, all three participants had different expectations for the advisor because the participants’ role perceptions were different. The students’ role perceptions of the advisor’s role influence students’ expectations of the advisor. Katherine’s independent style fitted well with her advisor’s hands-off approach, but Alice’s and Quinn’s styles did not. There is an assumption that Chinese culture has influenced Alice’s communication style. Alice’s high-context communication influenced by Chinese culture caused that the advisor was difficult to notice her expectation. Alice did not express her expectations might caused by implicit expression of requests for others in Chinese culture. This was only found from Alice’s case. Other two participants expressed that if they had requests, they tended to directly ask. Alice’s and Quinn’s expectations were influenced by their working experiences as professors in China. The role conflict between a doctoral student and a professor can influence Alice’s and Quinn’s expectations. The participants’ advisor did not or objectively can not notice the students’ different expectations, so the advisor did not fulfill Alice’s expectations. There was hierarchical academic norms in doctoral study. Quinn did not notice the advisor had more power, especially in the advisor’s own research. Quinn’s expectation of the advisor’s sharing information was difficult to be fulfilled when the advisor was not willing to share information. This was the reason why the three participants followed one advisor, however the advisor-advisee relationships were quite different. When students’ expectations were fulfilled/ not fulfilled, the advisor-advisee relationship tended to be positive/ negative. Thus, the fulfillment of students’ expectations can influence advisor-advisee relationships.

#### 5.1.2 Behaviors

Participants’ role perceptions influenced participants’ behaviors. The way individuals act is, to a large extent, determined by how they identify with their role, and this, therefore, influences their behavior (Hogg et al., 1995). Students’ behaviors can influence the advisor-advisee relationship. In this research, Alice’s behavior of asking for feedback without direct expression was influenced by her role perceptions, especially her role perceptions of the advisor’s role as a “general.” However, Alice’s indirect expression caused she did not to get feedback, which led to Alice’s ending the advisor-advisee relationship. There was power distance between the advisor and Alice. Alice did not consider that the advisor would grade her “F” with the power of a professor, which caused Alice’s delayed graduation. Quinn’s behavior of modifying experiment design was influenced by her role perception, mainly her the perception of her own role as a “co-researcher.” Working experience as a professor led to role ambiguity in Quinn’s case, which influenced Quinn’s behavior. Quinn did not notice that she did not have the power to modify the research design during the cooperation with the advisor. Quinn should discuss the collaborative norms with the advisor first, or at least, communicate with the advisor before action. Chinese culture of collectivism might influence Quinn’s behavior. When Quinn considered her modifying would benefit the research, the advisor, and herself, Quinn ignore her power in the research, because the behavior could be good for the team. This behavior of Quinn caused the advisor’s anger, and the advisor-advisee relationship was influenced. The hierarchical academic norms made the advisor consider Quinn’s behavior was offensive. The advisor can not fully understand Alice’s and Quinn’s intentions behind the behaviors, which also indirectly influenced the advisor-advisee relationships. Thus, students’ perceptions of their roles and the advisor’s role can influence students’ behaviors, which can influence advisor-advisee relationship.

To sum up, students’ role perceptions can influence their expectations of the advisor and their behaviors. Students’ unfulfilled expectations and some of the behaviors can influence the advisor-advisee relationship.

### 5.2 Research question 2: How do these engagements facilitate or hinder academic progression?

It was found in this research that the advisor-advisee relationship can influence doctoral students’ learning experience and graduation.

#### 5.2.1 Learning experience

Doctoral students’ learning experience is related to their mental health. In this study, Katherine’s good relationship with the advisor provided her positive learning experience and mental health. Alice and Quinn, who had poor relationships with the advisor, suffered during their study, and their learning experience was negative. Dai and Elliot (2022) suggested that both cultural adaptation and supervisors’ multicultural awareness are essential for supportive and inclusive research environments, preventing misunderstandings and enhancing learning experiences. In this study, the advisor might not aware Alice’s high-context communication and Quinn’s good intention for the team, and the advisor-advisee relationships were influenced. Eisenberg et al. (2009) found that students’ mental health can influence students’ academic progress. Positive advisor-advisee relationship is a kind of social support which can reduce students’ anxiety. It was aligned with Acoba’s (2024) study that social support is inversely associated with symptoms of anxiety and depression. The anxiety and depression during doctoral study can influence doctoral students’ emotional well-being. Therefore the advisor-advisee relationships can influence students’ learning experience, which can affect students’ mental health and then students’ academic progress.

#### 5.2.2 Graduation

One of the signs of doctoral students’ academic progress is graduation. In this study, Katherine graduated according to her schedule. Alice’s graduation was delayed because she changed advisors, which was caused by the negative advisor-advisee relationship. Alice notice the power distance between a professor and a doctoral student after she got an “F.” However, it can not change the fact that Alice’s graduation was delayed. Quinn’s graduation was delayed because of less communication between Quinn and her advisor, which was caused by the negative advisor-advisee relationship. The negative relationship caused less contact from the advisor, and Quinn was difficult to graduate. Quinn’s situation was mainly influenced by the hierarchical academic norms in doctoral study. Therefore, the advisor-advisee relationship can influence students’ graduation and then affect students’ academic progress.

In summary, the advisor-advisee relationship can influence doctoral students’ learning experience and graduation and further influence students’ academic progress.

## 6 Conclusion and limitation

This research explored how Ph.D. students’ role perceptions influenced advisor-advisee relationships and academic progress. The conclusion consists of the following three aspects. Firstly, Ph.D. students can have different perceptions of their roles and the advisor’s role. The different role perceptions are mainly influenced by their past experience. Secondly, the different perceptions of Ph.D. students’ roles and the advisor’s role lead to students’ different expectations of the advisor and different behaviors of the students. These expectations and behaviors can influence the advisor-advisee relationship. Thirdly, the advisor-advisee relationship can influence Ph.D. students’ academic progress by influencing students’ learning experience and graduation. Therefore, Ph.D. students’ role perceptions can influence the advisor-advisee relationship, and the advisor-advisee relationship can influence Ph.D. students’ academic progress.

While this was a case study in the English Department in a university, the application of this study is not limited to one department in one university. The reason why the researchers consider this study can apply in broader context is because individuals have similar or different role perceptions to influence their expectations and behaviors. Thus, doctoral students’ role perceptions can influence the expectations and behaviors as well, and further influence the advisor-advisee relationship. The students’ role perception is definitely not the only factor to influence the advisor-advisee relationship. However, the role perceptions can influence the advisor-advisee relationships together with other factors, such as culture conflicts or language barriers. The researchers consider that all the doctoral student should obviously or potentially have their perceptions of the advisors’ roles and their own roles. The variety of doctoral students’ role perceptions lead to students’ varied expectations and behaviors. The lessons about aligning expectations and understanding behaviors in advisor-advisee relationships could apply to other doctoral programs, such as other departments, universities. This study could also apply to both local student context and international student context due to the same reason that all the doctoral student should obviously or potentially have their perceptions of the advisors’ roles and their own roles. Thus, this study can apply to broader contexts to improve advisor-advisee relationships and students’ academic progress.

Based on the findings in this research, there are three suggestions for advising Ph.D. students. The first suggestion is that Ph.D. advisors need to try to identify expectations. For instance, advisors can try to communicate more about the students’ expectations by chatting with the students. Asking the questions like “do you think you are helped” or “what do you think I can do more to help you” would encourage students to express more. The second suggestion is that when the advisors find that students’ behaviors are not aligned with the advisor’s expectations or requests, the advisors need to try to communicate and negotiate with the students, which can also improve the advisor-advisee relationships and students’ academic progress. The third suggestion is paying more attention to culture differences. Culturally responsive advising practice will benefit for improving the advisor-advisee relationships.

There are three limitations to this study. First, all the interviewed participants were female students followed a male advisor. Gender differences may influence students’ role perceptions and the advisor-advisee relationships. Future studies can select interviewees depending on different genders of students followed different genders of advisors. Second, this research did not include the advisor’s role perceptions. The researcher tried to interview the advisor; however, the advisor refused. Future studies can try to interview students and their advisors, which would provide a complete view of role perceptions. Third, most of the collected data was based on the doctoral students who have already graduated. Thus, the dynamic, situated, or co-constructed process of the participants’ role perception construction was difficult to catch. Further study can try to choose the participants who just join the doctoral program, and follow up their the construction and negotiation of the role perceptions.

## Data Availability

The raw data supporting the conclusions of this article will be made available by the authors, without undue reservation.

## Appendix

### Semi-structured interview questions

Due to the reason that the interviews in this study were semi-structured interviews, some of the interview questions were generated from the participants’ answers. The researchers will only list the common interview questions for the three participants. The interview questions which are not related to the key concepts in this study, such as role perceptions, advisor-advisee relationships, and academic progress, will not be listed. The questions about the three participants’ basic information, such as name, age, profession, etc will not be listed either. Thus, the main semi-structured interview questions in this study are:

1. How was your advisor-advisee relationship?
2. What factors do you think influenced your advisor-advisee relationship?
3. Do you think there is culture conflict in your advisor-advisee relationship?
4. What was your basic expectations for the advisor? (When the advisor can not fulfill the basic expectations, the student would consider the advisor as not qualified.)
5. What were the advisor’s behaviors to fulfill/not fulfill your basic expectations?
6. What were your (common) behaviors when you got along with your advisor?
7. What were the advisor’s reactions toward your behaviors?
8. What roles do you think you and your advisor played in your advisor-advisee relationship? (For example, the advisor’s role could be a “gardener”, and your role could be a “plant”, something like this.)
9. How do you think your role and the advisor’s role established?
10. How did your advisor-advisee relationship influence your academic progress?
11. Would you please describe your feelings about your learning experiences?
12. Are you satisfied with your graduation time?
13. Why are you satisfied/not satisfied with your graduation time?
